# Tyloxapol inhibits ESX-1 secretion in *Mycobacterium marinum*

**DOI:** 10.1128/jb.00136-26

**Published:** 2026-04-22

**Authors:** Owen A. Collars, Simon D. Weaver, Richard L. Hernandez, Matthew M. Champion, Patricia A. Champion

**Affiliations:** 1 Department of Biological Sciences, University of Notre Dame 167056, Notre Dame, Indiana, USA; 2 Department of Chemistry and Biochemistry, University of Notre Dame 405050, Notre Dame, Indiana, USA; Dartmouth College Geisel School of Medicine, Hanover, New Hampshire, USA

**Keywords:** *Mycobacterium marinum*, tyloxapol, ESX-1, protein secretion

## Abstract

Mycobacteria have a hydrophobic cell envelope that makes uniform growth in liquid culture challenging. Non-ionic detergents, including Tween-80 and tyloxapol, are commonly added to media when culturing mycobacterial species in the laboratory. Tyloxapol was reported to exhibit anti-tuberculous activity during animal infection with *M. tuberculosis* in the 1950s. In the 1980s, microscopy studies suggested that tyloxapol impacted the interaction between *M. tuberculosis* and the phagosomal membrane, preventing mycobacterial access to the cytoplasm. It is now known that the ESX-1 Type VII secretion system mediates the interaction between pathogenic mycobacteria and the phagosomal membrane. *Mycobacterium marinum* is a pathogenic mycobacterial species that has been widely used to understand the molecular mechanisms and host responses to the ESX-1 system. The hemolytic activity of *M. marinum* allows the study of ESX-1 lytic activity outside of the context of a host cell. We found that tyloxapol inhibits the hemolytic activity of *M. marinum* in a concentration-dependent manner. We discovered the mechanism underlying the inhibition of hemolysis is the differential inhibition of the production and secretion of ESX-1 substrates. Proteomics analysis indicates that tyloxapol specifically inhibited the secretion of a subset of ESX-1 substrates in addition to specific PE/PPE/PEGRS proteins, but not the secretion of proteins secreted by the Sec, ESX-3, or ESX-5 secretion systems. Our findings directly impact how the field interprets data from studies where *M. marinum*, and potentially other mycobacterial species, were grown in tyloxapol. Our findings may explain the original observations linking tyloxapol to anti-tuberculosis activity.

**IMPORTANCE:**

Tuberculosis, which is caused by *Mycobacterium tuberculosis*, is one of the world’s deadliest diseases. We lack a clear understanding of how *M. tuberculosis* and related mycobacterial species cause disease. In the 1950s, it was reported that treating *M. tuberculosis*-infected animals with tyloxapol improved the survival and, in some cases, protected the animals from death. Tyloxapol is a detergent that is commonly added to mycobacterial cultures to promote dispersed growth in the laboratory. Later studies suggested that tyloxapol altered the interaction between *M. tuberculosis* and the phagosomal membrane during macrophage infection. The ability to escape the phagosome is essential for mycobacteria to cause disease and is mediated by the ESX-1 Type VII protein secretion system. Using *M. marinum*, a well-established model for understanding the molecular mechanisms of ESX-1 secretion, we show that tyloxapol used at more than 100-fold less than what is commonly used to grow mycobacteria in the lab inhibits ESX-1 secretion. Our findings have widespread implications on how we interpret our findings as a field and may explain why tyloxapol impacted *M. tuberculosis* infection of both animals and macrophages. Our study also indicates that tyloxapol can be used as a tool to understand the molecular mechanisms of ESX-1 protein secretion.

## INTRODUCTION

Early during macrophage infection, mycobacterial pathogens reside within and adapt to the phagosome (1–3). The disruption of the phagosomal membrane allows bacterial interaction with the cytoplasm (4, 5). Cytoplasmic access initiates the host response to infection and macrophage lysis, which is required for disease progression (4, 6–8).

The mycobacterial cell envelope is hydrophobic (9). As such, the non-ionic detergents Tween-80 and tyloxapol are routinely used in culturing mycobacterial cells to prevent aggregation during *in vitro* growth. In the 1950s, tyloxapol was reported to exhibit anti-tuberculous activity during animal infection with *M. tuberculosis* (10–13). Microscopy-based studies in the 1980s proposed that tyloxapol impacted the interaction between *M. tuberculosis* and the phagosomal membrane, preventing mycobacterial access to the cytoplasm (14, 15). The mechanisms used by *M. tuberculosis* to interact with the phagosome were unknown when these studies were published.

Type VII secretion systems promote mycobacterial and Gram-positive pathogenesis and physiology (16). ESX-1 is a conserved Type VII system in *M. tuberculosis* and in *M. marinum* (17–20). *Mycobacterium marinum* is a mycobacterial pathogen that causes a tuberculosis-like infection of ectothermic animals (21). *M. marinum* is a widely accepted model for understanding mycobacterial pathogenesis, and in particular for studying ESX-protein secretion (22, 23). It is well established that the *M. marinum* ESX-1 system is essential for phagosome escape and translocation to the cytoplasm (4–7). Stains lacking the ESX-1 system are retained in the phagosome and attenuated (6, 24, 25). Additional ESX-secretion systems function downstream of the ESX-1 system, including ESX-3 and ESX-5 (26, 27). The ESX-3 system secretes proteins that prevent repair of the phagosome and are important for metal homeostasis (26, 28–32). The ESX-5 system secretes protein toxins and the majority of the PE/PPE/PE-PGRS proteins and is required for glucose and glycerol uptake (33–41).

Based on the role of ESX-1 in phagosomal lysis and the microscopy studies suggesting that tyloxapol impacted the interaction between *M. tuberculosis* and the phagosomal membrane (14, 15), we hypothesized that tyloxapol directly inhibited ESX-1 activity.

## RESULTS

### Tyloxapol inhibits hemolytic activity in *M. marinum* in a concentration-dependent manner

**Fig 1:**
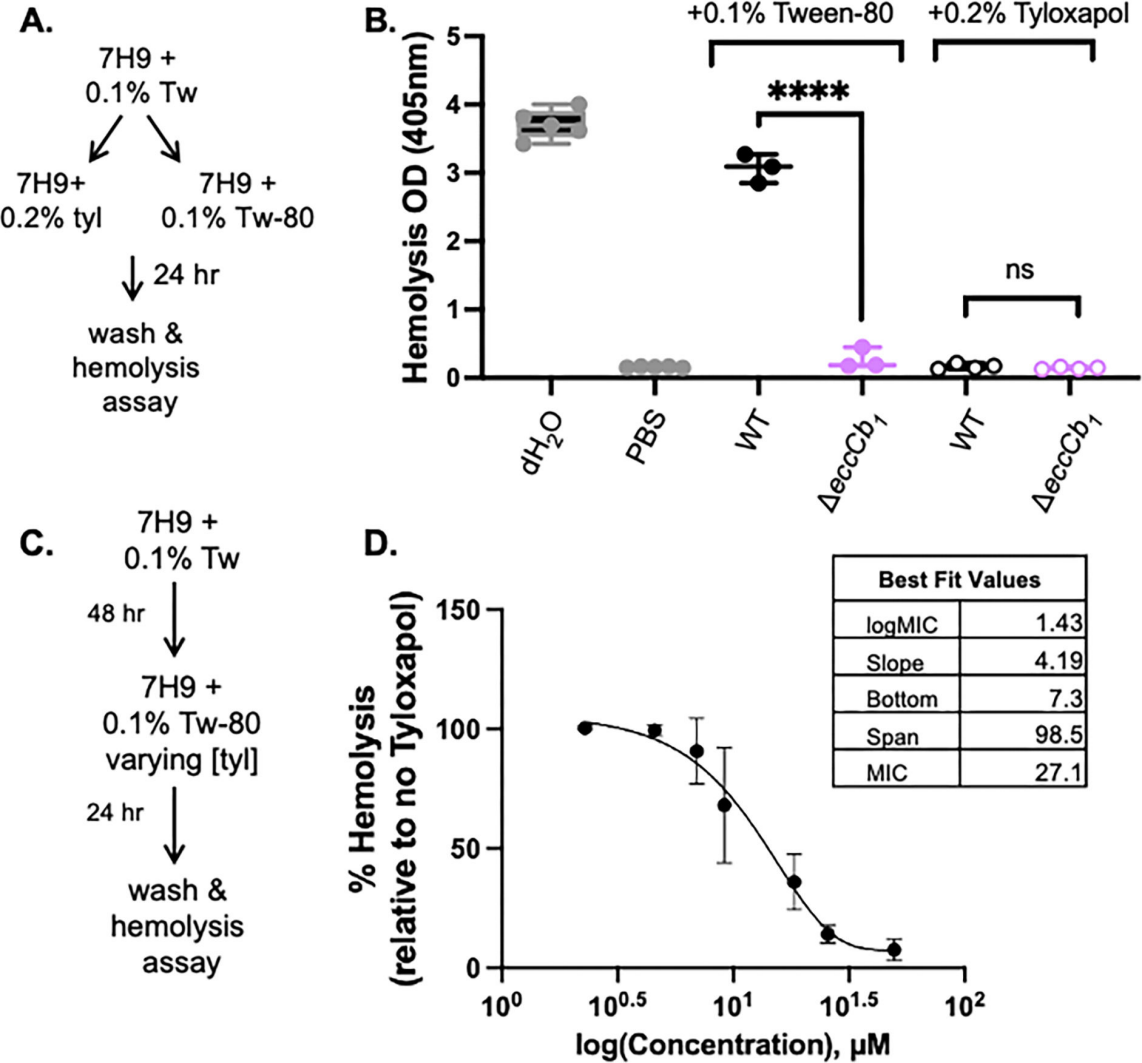
Tyloxapol inhibits ESX-1-dependent hemolytic activity in a concentration-dependent manner. (**A**) Schematic of growth conditions for panel B. (**B**) Hemolytic activity of *M. marinum* grown in 0.1% Tween-80 or 0.2% tyloxapol for 24 h. Each data point represents a biological replicate consisting of three technical replicates. Significance was determined using a one-way ordinary ANOVA (*P* < 0.0001), followed by a Tukey’s multiple comparison test ****P < 0.0001. (**C**) Schematic of growth conditions for panel D. (**D**) Tyloxapol inhibition curves of hemolytic activity. Bacteria were grown in the presence of 0.1% Tween-80 with varying amounts of tyloxapol from 0.000625% through 0.01355%. Individual data points represent the average of three independent experiments. Error bars are the standard deviation. MIC was calculated using a Gompertz equation for MIC determination. Tyloxapol concentration was determined using an estimated molecular weight of 3,000 g/mol, which is the median distance between the upper and lower bounds of the polymer lengths reported by Sigma and Santa Cruz Chemicals.

*M. marinum* lyses red blood cells in a contact-dependent, ESX-1-dependent manner (20). The hemolytic activity of *M. marinum* is a major strength of this model because it allows the synchronous ESX-1 lytic activity in the absence of a host cell. To test if growth in tyloxapol impacted hemolytic activity, we grew the WT and Δ*eccCb_1_ M. marinum* strains in 7H9 media with 0.1% Tween-80 (Fig. 1A). EccCb_1_ is a conserved cytoplasmic component of the ESX-1 system that is required for ESX-1 secretion (17, 18, 42, 43). We washed the cells and cultured the strains in 7H9 media with either 0.1% Tween-80 or 0.2% tyloxapol at pH 6.8 for 24 h. The cells were washed to remove the detergents and the buffered media, and then resuspended in PBS to perform the hemolysis assays. As shown in Fig. 1B, the WT *M. marinum* strain was significantly more hemolytic than the Δ*eccCb_1_* strain following growth with Tween-80 at pH 6.8, consistent with previously published findings (20, 43, 44). However, there was no significant difference in the hemolytic activity of the WT and the Δ*eccCb_1_* strains following growth with tyloxapol at pH 6.8. From these data, we conclude that growth in tyloxapol inhibited the ESX-1-dependent hemolytic activity of *M. marinum*.

We next tested if the inhibition of hemolysis by tyloxapol was concentration dependent. We grew *M. marinum* in 7H9 buffered to pH 6.8 in 0.1% Tween-80 with varying concentrations of tyloxapol for 24 h, washed the cells, and performed hemolysis assays (Fig. 1C). As shown in Fig. 1D, the inhibition of *M. marinum* hemolytic activity was concentration dependent. We did not notice altered growth of *M. marinum*, indicating that the inhibition of hemolytic activity was not due to inhibition of *M. marinum* growth. The best-fit value for the MIC of tyloxapol sufficient to block *M. marinum* hemolytic activity was 27.1 μM (95% CI, 18.6–46.2 μM).

### Tween-80 catabolism is not a signal for hemolytic activity

**Fig 2:**
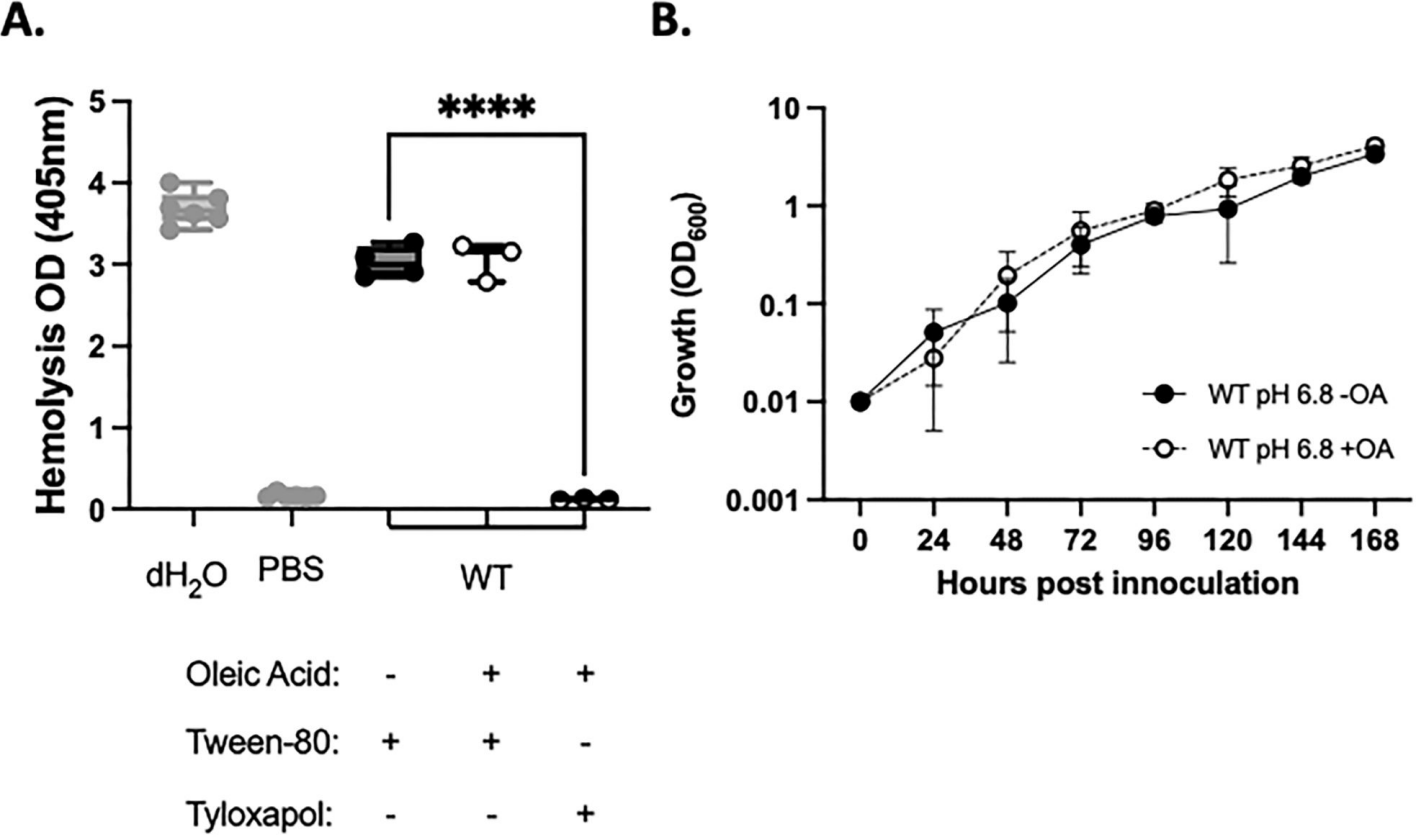
Oleic acid is not a hemolysis-stimulating signal. (**A**) Hemolytic activity of *M. marinum* grown in with 0.1% Tween-80 or 0.2% tyloxapol with or without 200 mM oleic acid for 24 h. Each data point represents a biological replicate each including three technical replicates. Significance was determined using a one-way ordinary ANOVA (*P* < 0.0001), followed by a Tukey’s multiple comparison test comparing each sample to the WT strain grown in 0.1% Tween-80. ****P < 0.0001. (**B**) WT *M. marinum* strains in 7H9 media with 0.2% tyloxapol. Strains were grown for 7 days, with growth readings taken every 24 h by OD_600_. Statistical analysis using a two-way ANOVA, followed by a Tukey’s multiple comparison test. The two curves are not significantly different. The *y*-axis is log_10_ scale.

Tween-80 is catabolized by mycobacteria into oleic acid, which can be used as carbon source (45–47). Tyloxapol is not catabolized by mycobacteria (48), making tyloxapol an attractive detergent for studies focused on metabolism. To rule out that the oleic acid catabolite promoted hemolysis, we supplemented cultures with 200 μM oleic acid and measured hemolytic activity. The addition of oleic acid did not significantly impact the hemolytic activity of the WT strain under any condition tested (Fig. 2A). We likewise found that the addition of oleic acid did not impact *M. marinum* growth under the conditions tested (Fig. 2B). From these data, we conclude that the oleic acid product due to Tween-80 catabolism is not a hemolysis-promoting signal for *M. marinum* under these growth conditions.

### Tyloxapol differentially impacts ESX-1 substrate production and secretion

We sought to identify the mechanism underlying the inhibition of *M. marinum* hemolysis following growth in tyloxapol. We reasoned that growth in tyloxapol may be impacting the production or secretion of ESX-1 substrates from *M. marinum*.

**Fig 3:**
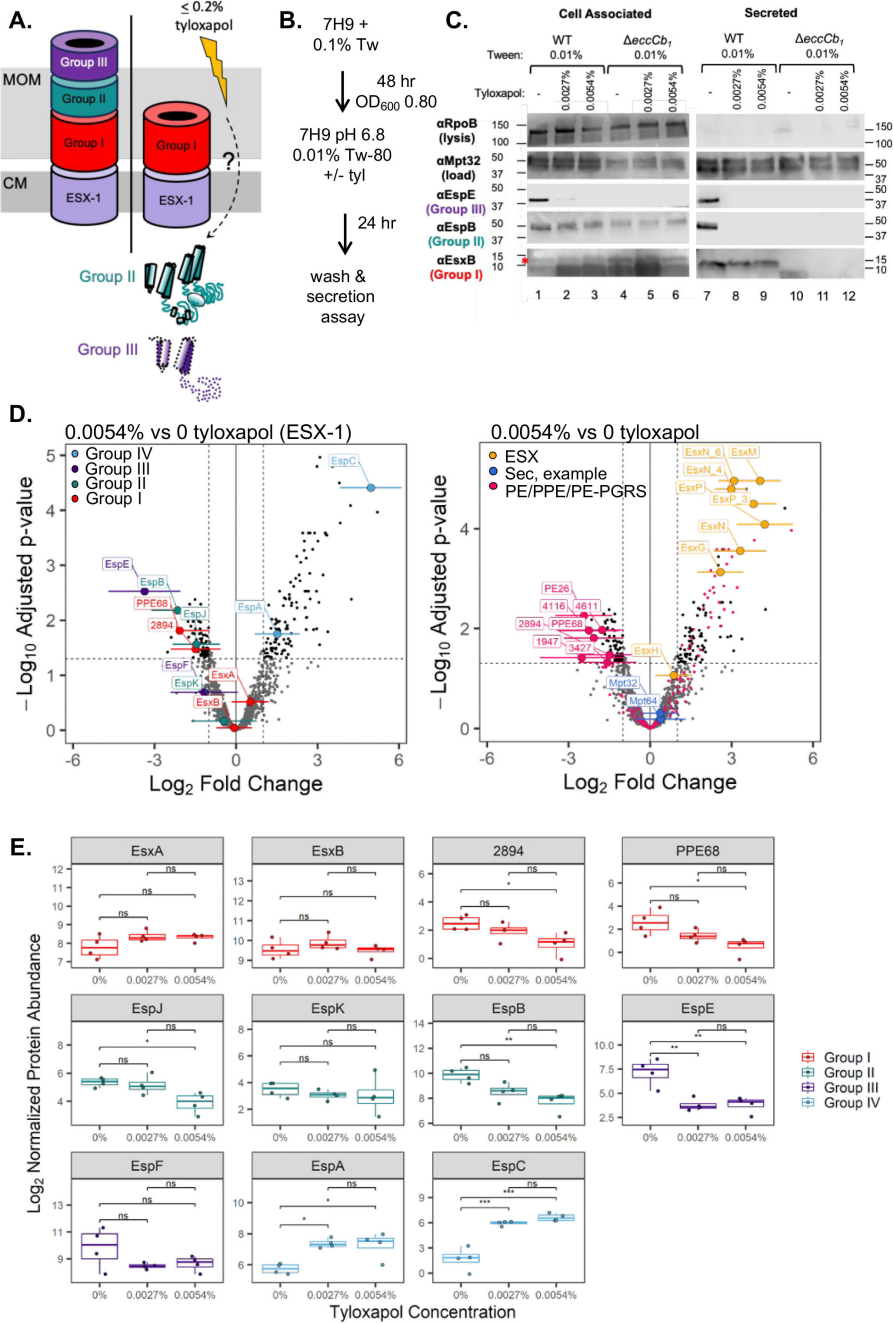
Tyloxapol differentially affects the production and secretion of ESX-1 substrates in *M. marinum*. (**A**) Schematic of ESX-1 secretion assembly. CM = cytoplasmic membrane. MOM = mycolate outer membrane. ESX-1: ESX-1 conserved components, including EccA1, EccB_1_, EccCa_1_, EccCb_1_, EccD_1_, EccE_1_, and MycP_1_. Group I, II, and III substrates are described in the text, as designated in Cronin et al. We propose that tyloxapol is sensed by the mycobacterial cell by an unknown mechanism. Our data support that Group II substrates are made, but not secreted, while the Group III substrates are not made or secreted. (**B**) Schematic of growth conditions for panel C. (**C**) Secretion assay in the presence of 0.0027 and 0.0054% tyloxapol. 30 μg of protein was loaded in each lane. Immunoblots are representative of at least three biological replicates. MPT-32 is a protein secreted by the Sec secretion system and is a loading control for both cell-associated and -secreted protein fractions. RpoB is a component of RNA polymerase. RpoB is a loading control for the cell-associated fraction and a lysis control for the secreted protein fraction. The EsxB band is marked by an asterisk in lanes 1–6. (**D**) Volcano plots showing change in protein abundance as measured by label-free quantitative proteomics in the secretome with treatment growth at 0.0054% tyloxapol compared to 0% in *M. marinum* (*n* = 4 biological replicates). Log_2_ fold change (LFC) plotted on the *x*-axis (positive values indicate higher in 0.0054% tyloxapol, while negative values indicate higher in 0% tyloxapol) and −Log_10_ Benjamini-Hochberg (B-H) corrected *P*-value plotted on the *y*-axis. Error bars show 95% confidence intervals of LFC. Cutoffs (dotted lines) set at B-H *P*-value of 0.05 and absolute LFC of 1. Left panel shows ESX-1 substrates colored by secretion group (red = I, green = II, purple = III, blue = IV). Right panel shows ESX-3 and ESX-5 substrates (yellow), example Sec proteins (blue), and PE/PPE family proteins (pink) with the PE/PPE family proteins of significantly lower abundance in the 0.0054% tyloxapol sample labeled. (**E**) Box and whisker plots showing change in protein abundance as measured by label-free quantitative proteomics in the secretome for the ESX-1 substrates highlighted in (**D**) across three concentrations of tyloxapol (0, 0.0027, and 0.0054%). Each point is a biological replicate. Statistical significance was calculated at the proteome level using the limma empirical Bayesian approach and with a B-H multiple hypothesis testing correction applied. Ns = not significant, * = B-H adj. *P*-value < 0.05, ** = B-H adj. *P*-value < 0.01, *** = B-H adj. *P*-value < 0.001. Proteins colored by group as in (**D**).

We previously published that ESX-1 substrates are secreted in a hierarchy under standard laboratory conditions (43). Three groups of substrates undergo ordered secretion by the ESX-1 membrane complex (Fig. 3A, light purple). Group I substrates, including the PPE68/MMAR_2894 and EsxA/EsxB (ESAT-6/CFP-10) substrate pairs (red, Fig. 3A), are required for groups II (EspB/EspK pair and EspJ, teal) and III (EspE/EspF pair, purple) substrate secretion. Group II substrates are required for Group III substrate secretion. Group III substrates are not required for ESX-1 substrate secretion, but are essential for hemolytic activity and virulence in a macrophage model of infection (43, 44). Because the Group I and II substrates are essential for the secretion of the Group III substrates, they are also essential for hemolysis (43).

To test how tyloxapol impacted the production and secretion of the ESX-1 substrates, we sought to mimic the media conditions from the hemolysis assay in Fig. 1. Secreted proteins have historically been isolated from *Mycobacterium* grown in Sauton’s medium (17, 20, 49, 50). We tested if ESX-1 secretion could be detected in the presence and absence of tyloxapol following growth in 7H9. As shown in Fig. 3B, EsxB (Group I), EspB (Group II), and EspE (Group III) were made (lane 1) and secreted from the WT strain (lane 7) grown in 7H9 media supplemented with 0.01% Tween-80. Addition of 0.0027% or 0.0054% tyloxapol to 7H9 supplemented with 0.01% Tween-80 differentially affected the production and secretion for the ESX-1 substrates. Notably, EsxB was produced (lanes 2 and 3) and secreted from the WT strain (lanes 8 and 9) in the presence of tyloxapol. Although EspB was produced (lanes 2 and 3), EspB was not secreted from the WT strain grown in tyloxapol (lanes 8 and 9). Growth in tyloxapol resulted in reduced levels of EspE produced in the WT strain (lanes 2 and 3) and a loss of detectable EspE secretion (lanes 8 and 9). EspE was not detected in the cell-associated fraction of the Δ*eccCb_1_* strain due to transcriptional feedback control (51–53). EsxB, EspB, and EspE were not secreted from the Δ*eccCb_1_* strain. From these data, we conclude that tyloxapol differentially affected the production of the EspB (Group II) and EspE (Group III) substrates and resulted in a loss of EspB and EspE secretion, likely providing a mechanistic explanation for the loss of ESX-1-dependent hemolytic activity of *M. marinum* following growth in tyloxapol.

To determine if tyloxapol inhibits the secretion of additional proteins from *M. marinum*, we measured the levels of protein secretion from wild-type *M. marinum* grown in 7H9 supplemented with 0.01% Tween-80 with no tyloxapol or with the addition of 0.0027 or 0.0054% tyloxapol, as Fig. 3B. We used label-free quantification (LFQ) mass spectrometry proteomics to measure the relative changes in the levels of protein secretion. We measured 1731 proteins in the secreted fractions (1% false discovery rate). Growth in 0.0027 or 0.0054% tyloxapol resulted in significant reductions in the abundance of 17 and 71 proteins in the secreted protein fractions, respectively (Data set S1). Notably, as shown in Fig. 3D, left panel, and Fig. 3E, growth in 0.0054% tyloxapol significantly reduced the secretion of Group III substrate (EspE, purple), Group II substrates (EspB, EspJ, teal), and Group I substrates (PPE68 and MMAR_2894, red) from *M. marinum*. Additional substrates from each of these groups were unaffected, including EsxB, which is aligned with the immunoblot analysis in Fig. 3C and E. Conversely, the Group IV substrates EspA and EspC showed a significant increase in secretion following growth in tyloxapol. We also measured a significant reduction in the secretion of three PE proteins (MMAR_1538/PE26, MMAR_4611, and MMAR_3427), one PPE protein, MMAR_1947, and one PE-PGRS protein, MMAR_4116). The secretion of MPT-32 and MPT-64, both substrates of the Sec system, was unaffected (Fig. 3D, right panel). Substrates of the ESX-3 and ESX-5 systems, additional Esx proteins, and 31 proteins from the PE, PPE, and PE-PGRS families were present at higher levels in the secreted fraction following growth of *M. marinum* in 0.0054% tyloxapol (Fig. 3D, right). From these data, we conclude that the inhibitory effect on protein secretion is specific to a subset of the group I, II, and III ESX-1 substrates and specific PE/PPE/PE/PGRS proteins.

## DISCUSSION

In this study, we showed that tyloxapol at levels 100–200× lower than are commonly used in mycobacterial culturing differentially blocks the production and secretion of specific ESX-1 substrates from *M. marinum*, abrogating hemolytic activity. It is well established that the secretion of the Group II and III substrates, which are impacted by tyloxapol, is essential for mycobacterial virulence (20, 43, 44, 54–56). We and others have shown that deletion of the ESX-1 system in *M. marinum* or in *M. tuberculosis* alters transcription (51, 57). It is unclear if tyloxapol inhibits the ESX-1 system in other mycobacterial species.

Our proteomics data suggest that tyloxapol does not inhibit the secretion of proteins from the Sec secretion system or the paralogous ESX-3 and ESX-5 secretion systems. Instead, our data indicate that tyloxapol specifically inhibits secretion of proteins by the ESX-1 system, underscoring that this is not a generalized detergent effect. The levels of substrates from the ESX-3 and ESX-5 systems and numerous additional PE/PPE/PE-PGRS proteins were increased in the secreted protein fraction, which is reminiscent of our findings in prior studies where ESX-1 secretion was inhibited (43, 58, 59). PE, PPE, and PE-PGRS family proteins have been implicated in solute and, potentially, protein transport in the mycolate outer membrane (33–37). Interestingly, in addition to PPE68 and MMAR_2894, which are known ESX-1 substrates, only five of these proteins exhibited significantly reduced secretion following growth in tyloxapol. Our findings may indicate that these proteins are either secreted by or colocalized in the envelope with the ESX-1 system in *M. marinum*.

Tyloxapol may have additional impacts on mycobacterial physiology, which may alter how we interpret our work as a field. Detergents are widely used in culturing mycobacterial species in the laboratory. At the time of this manuscript, 223 publications were found in PubMed Central when “*Mycobacterium marinum* and tyloxapol” were searched together, with 31 in 2025 alone. Similar searches with other mycobacterial species yielded 1,154, 747, and 206 publications for *M. tuberculosis*, *M. smegmatis*, and *M. abscessus* and tyloxapol, respectively. The studies that use tyloxapol for growth focus on a range of topics, from fundamental physiology to pathogenesis. Tyloxapol is also exclusively used in media preparations aimed at maintaining virulence lipid production during laboratory growth of *Mycobacterium* (60). It is important as a field to consider how growth in tyloxapol and its impacts on ESX-1 alter how we interpret our findings, especially when considering pathogenesis mechanisms.

Under the conditions tested in this study, the addition of oleic acid did not impact hemolytic activity. It is possible that other products of Tween-80 catabolism, for example, palmitic, linoleic, or stearic acids, could be signals for hemolytic activity. Alternatively, it is possible that we did not include the appropriate concentration of oleic acid to mimic Tween catabolism during mycobacterial growth. Further studies need to be performed.

Our work may provide a mechanism to explain earlier literature linking tyloxapol to anti-tubercular activity. Beginning in the 1950s, several independent groups published data supporting that tyloxapol (also named Macrocyclon, Triton WR-1339) is protective against *M. tuberculosis* infection (10–12, 61). Although tyloxapol is not bactericidal, adding tyloxapol to the growth media of primary macrophages inhibited *M. tuberculosis* growth during infection (62). Likewise, macrophages isolated from tyloxapol-treated mice were more resistant to *M. tuberculosis* infection. Treating mice with tyloxapol prior to or following *M. tuberculosis* infection was protective against death. In fact, the CFUs from lungs and spleens and time to death from mice infected with *M. tuberculosis* in the presence of tyloxapol were similar to infections with the attenuated BCG vaccine strain (63, 64), which has a natural deletion of several ESX-1 genes (19, 25). The addition of tyloxapol before or after infection of guinea pigs and mice reduced infection or even healed infected animals (13). Microscopy data from the 1980s suggested that tyloxapol altered the interaction between *M. tuberculosis* and the phagosome membrane (14). It is now well established that the ESX-1 system is essential for phagosomal lysis (4, 6). Our studies may provide a mechanism by which tyloxapol blocks the production and secretion of ESX-1 substrates required for phagosomal lysis during macrophage infection. It is unclear how long the inhibition of ESX-1 secretion due to growth in tyloxapol lasts. However, it has been suggested that growth of mycobacteria in Tween-80 changes the host response to infection relative to detergent-free cultured bacteria (46, 47, 65). Further experiments are required to determine if growing mycobacteria in tyloxapol impacts *in vivo* infection.

Historically, mycobacterial short-term culture filtrates (STCFs) have been generated from cultures grown in Sauton’s medium (49). In *M. smegmatis*, ESX-1 substrates were detected in STCFs from strains grown in Sauton’s medium, but not from 7H9 medium (50). Our study indicates that ESX-1 proteins from *M. marinum* can be detected from STCFs generated from cultures grown in 7H9 medium. It is possible that the inability to secrete proteins in 7H9 is specific to *M. smegmatis*.

Tyloxapol may be a new tool to study the mechanics of ESX-1 secretion. We demonstrate that tyloxapol blocks ESX-1 activity *in vitro* by impacting the secretion of the group II and III substrates, but not Group I substrates, from *M. marinum*. Tyloxapol could affect targeting or translocation of the Group II substrates, effectively stopping ESX-1 from switching from the Group I substrates to secreting the Group II substrates and beyond. The reduced levels of EspE could indicate a feedback mechanism in response to a loss of Group II secretion. Interestingly, tyloxapol may promote switching between ESX-1 substrate groups (66). The Group IV ESX-1 substrates, EspA and EspC, showed a significant increase in secretion following growth in tyloxapol. These findings align with our previous work that indicated increased secretion of Group IV substrates in strains that failed to secrete the Group II/III substrates (43). It is unclear how *M. marinum* senses tyloxapol in the media and transmits the signal to the cytoplasm, where substrate production and translocation are being impacted. Current studies are focused on identifying the pathways that sense tyloxapol and the response that disrupts ESX-1 secretion.

## MATERIALS AND METHODS

### Growth and generation of bacterial strains

The *M. marinum* M (ATCC BAA-535) and Δ*eccCb_1_* strains (42) were maintained in Middlebrook 7H9 broth (Sigma-Aldrich, St Louis MO) with 0.5% glycerol and 0.1% Tween-80 (Fisher Scientific, Pittsburgh PA) or on Middlebrook 7H11 agar (Sigma-Aldrich) with 0.5% glycerol and 0.5% glucose at 30°C. To buffer the pH of the liquid media, 7H9 broth was supplemented with 100 mM 3-(*N*-morpholino)propanesulfonic acid (MOPS) (Thermo Fisher, Waltham, MA) and buffered to pH 6.8 (7H9 pH6.8). Tyloxapol (Chem-Impex, Wood Dale IL) was added as indicated.

### Hemolysis assay

*M. marinum* was grown in 7H9 broth with 0.1% Tween-80 to mid-log phase. Then, 24 h prior to the hemolysis assay, the bacteria were sub-cultured into 7H9 pH 6.8 overnight. Where applicable, varying concentrations of tyloxapol as indicated in the figures and text or 200 μM oleic acid (Thermo Fisher, Waltham, MA) was added to cultures. Hemolysis assays were performed exactly as in reference 43.

### ESX-1 secretion assay

*M. marinum* strains were grown in 7H9 media supplemented with glucose, glycerol, and 0.1% Tween-80 until turbid for ~5 days. The cultures were diluted to an OD_600_ of 0.8 in 50 mLs of 7H9 pH 6.8 with 0.01% Tween-80 in the presence and absence of tyloxapol as indicated. The cells were grown for 48 h at 30°C. The culture supernatants and cell-associated fractions were prepared as described in reference 43, except that, following protein quantification, proteins were precipitated in four times the volume of acetone (Thermo Fisher) for 1 h. Samples were centrifuged at 14K rpm for 10 min at 4°C. Acetone was removed, and protein samples were air-dried and resuspended in 16 μL of PBS prior to SDS-PAGE. Next, 20 µg of protein was loaded onto 4–20% TGX Gradient Gels (Bio-Rad) and separated by gel electrophoresis.

### Immunoblotting analysis

Immunoblots were performed as in reference 67, except they were imaged using a Licor C-Digit digital developer (LICORbio, Lincoln NE) and analyzed using Image Studio 6.1 (LICORbio). The following reagents were obtained through BEI Resources, National Institute of Allergy and Infectious Diseases (NIAID), National Institutes of Health (NIH): polyclonal anti-*Mycobacterium tuberculosis* Mpt32 (*Rv1860*, antiserum, rabbit) NR-13807 (1:20,000) and polyclonal anti-*Mycobacterium tuberculosis* CFP10 (*Rv3874*, antiserum, rabbit) NR-13801 (1:5,000). The following antibodies were generated by GenScript (Piscataway, NJ): the EspE antibody (1:5,000 dilution, rabbit, epitope: CGQQATLVSDKKEDD) based on reference 56 and the EspB antibody (1:1,000 dilution, rabbit, epitope: TKADLEPVNPPKPP) based on reference 54.

### Proteomics/LC-MS

Secreted fractions from culture filtrates were prepared for LC-MS analysis via S-trap digestion, as described (66, 68) in biological quadruplicate with the following modifications: 25 μg of each sample was precipitated and stored in acetone (J.T. Baker, Radnor, PA) at −20°C for 5–7 days. Samples were dried and prepared in 140 mM triethylammonium bicarbonate (TEAB, Millipore-Sigma, St. Louis, MO), 10% sodium dodecyl sulfate (SDS, Millipore-Sigma), and 10 mM tris(2-carboxyethyl)phosphine (TCEP, Millipore-Sigma). Samples were heated for 10 min at 95°C and cooled, and then iodoacetamide (IAA, MP Biomedicals, Solon, OH) was added to 18 mM for 30 min in the dark.

Samples were acidified with o-phosphoric acid (Millipore-Sigma) to a final concentration of 1.1% in 60 μL, and then flocculated with 382 μL binding buffer containing 90% methanol (J.T. Baker) and 10% 1M TEAB. Tryptic digestion (Trypsin Gold, Promega, Madison, WI) on S-Trap micro devices (Protifi, Huntington, NY) and desalting with HLB SPE columns (hydrophilic-lipophilic balance solid-phase extraction, Waters, Milford, MA) were performed as previously described (66, 68).

Desalted peptides were resuspended in 0.1% formic acid (Millipore-Sigma) and water (J.T. Baker) to a concentration of 1 mg/mL. Next, 500 ng of sample was injected in technical duplicate onto a nanoElute 2 and timsTOF Pro 2 LC-MS system. LC methods were as described, with samples loaded in 2 μL injections of 250 ng/μL (66). Nano-ESI was used with a spray voltage of 1,700 V. MS was set to the parallel accumulation, serial fragmentation data-independent mode (PASEF-DIA), and instrument tune parameters were set to default for proteomic studies as previously described (66). Raw data files and instrument parameters were uploaded to massIVE and Proteome Xchange, with details found in the data availability section below.

### Data analysis

Bruker data files (.d) were searched using Spectronaut (v19.2.240905.6263) with directDIA analysis using the *Mycobacterium marinum (strain ATCC BAA-535/M*) reference proteome (UP000001190_216594; 5418 entries) downloaded on 24 November 2025. BGS factory settings were used. The cleavage rules were set to trypsin/*P* with two allowed missed cleavages. Carbamidomethyl (C) was set as a fixed modification, and acetyl (protein N-term), deamidation (NQ), and oxidation (M) were set as variable modifications. Protein results were exported as .tsv files, which can be found in the massIVE data submission as described in the data availability section below.

Data were analyzed in R (v4.3.1) as described previously (66). Briefly, technical duplicates were combined by averaging to produce a single quantitative value (log_2_ transformed) for each protein for each biological replicate. The remaining analysis was performed using these values (*n* = 4 per tyloxapol concentration). Biological replicates were median normalized with QFeatures (v1.10.0), and differential expression analysis between the different tyloxapol concentrations (0, 0.0027, and 0.0054%) was performed with the limma (v3.56.2) empirical Bayesian method (69). Multiple hypothesis testing correction was performed with the Benjamini-Hochberg (B-H) method built into the limma package. Volcano plots were created by plotting –Log_10_ of this corrected *P-*value against the Log_2_ fold change (LFC) with visual cutoffs set at LFC of –1 and 1 and a significance cutoff at <0.05 B-H adjusted *P-*value. Specific proteins of interest were pulled out of this data set (e.g., ESX substrates) and plotted as box and whisker plots showing each biological replicate as one point, but annotated with the statistical significance from B-H adjusted *P*-values as described above (ns = not significant, * = adj. *P-*value < 0.05, ** = adj. *P-*value < 0.01, *** = adj. *P-*value < 0.001). The data set is included as a supplemental file.

## Data Availability

Raw data files, search parameters, database, and instrument method parameters were deposited in massIVE (identifier MSV000100929) and Proteome Xchange (identifier PXD074724).

## SUPPLEMENTAL MATERIAL

Data set S1 jb.00136-26-s0001.xlsx Each sheet is a different comparison between tyloxapol levels. Null = 0%, low = 0.0027%, and high = 0.0054%.

ASM does not own the copyrights to Supplemental Material that may be linked to, or accessed through, an article. The authors have granted ASM a non-exclusive, world-wide license to publish the Supplemental Material files. Please contact the corresponding author directly for reuse.
